# Factors associated with increased risk of postoperative blood transfusion in patients undergoing total hip arthroplasty at an Irish University Hospital

**DOI:** 10.1007/s11845-024-03653-1

**Published:** 2024-03-12

**Authors:** Gavin David O’Connor, Stephen Mannion, James Purcell

**Affiliations:** 1 School of Medicine, University College Cork, Cork, Ireland; 2 Department of Anaesthesiology, South Infirmary-Victoria University Hospital, Cork, Ireland

**Keywords:** Anaesthesia, Anaesthesiology, Blood transfusion, Orthopaedic surgery, Postoperative blood transfusion, Preoperative anaemia, Total hip arthroplasty

## Abstract

**Introduction:**

Approximately 7000 total hip arthroplasty (THA) surgeries occur in Ireland each year. A number of preoperative factors have been identified that increase the risk of postoperative blood transfusion after THA, including anaemia. The ability to identify patients at risk may allow preoperative management strategies to reduce blood transfusions. Data from Irish orthopaedic patients is currently lacking.

**Aim:**

To investigate if preoperative anaemia and other factors are associated with postoperative blood transfusions in patients who undergo THA.

**Methods:**

A retrospective cohort study of all patients who underwent THA in 2019 in SIVUH, Cork, using medical chart review.

**Results:**

In total, 350 charts met the inclusion criteria, with 291 charts reviewed. 8.9% of the patients who underwent THA had preoperative anaemia. Among these, 19.2% had a postoperative blood transfusion, compared to 1.5% of patients who were not anaemic preoperatively. The odds of receiving a blood transfusion was 15.5 times greater in the preoperative anaemia group compared to the non-anaemic group. Increasing age and higher ASA scores were associated with preoperative anaemia and postoperative blood transfusions. Length of stay was increased by 2.2 days (*p* < 0.00016) if blood transfusion was required.

**Conclusion:**

Preoperative anaemia was common in an Irish orthopaedic population undergoing THA. Preoperative anaemia predisposes patients to the greatest increased risk of postoperative blood transfusions. The other factors associated with the need for postoperative transfusion were ASA grade 3 or more and age greater than 65 years. Patients who received postoperative blood transfusions had a significantly increased length of hospital stay.

## Introduction

Total hip arthroplasty (THA) is very common in Ireland and internationally, with an estimated 7000 surgeries occurring annually in Ireland [1] and 332,000 performed in the US in 2010 [2]. It is performed by inserting a prosthetic joint into the proximal part of the femur and augmenting the surrounding structures to fix the prosthesis [3]. It is a common treatment for hip problems such as osteoarthritis [4]. The typical patient who undergoes THA is elderly (over 60). With our ageing global population, we would expect THA to become even more common in Ireland.

The W.H.O. defines anaemia as a haemoglobin level of < 13 g/dl for men and < 11 g/dl for women [5]. Anaemia is a common condition that increases in prevalence with age and affects the elderly population, with some studies finding 20% of people over 64 being anaemic [6]. This demographic is the most likely to undergo THA, and therefore, anaemia is an important factor to be aware of preoperatively, with some studies finding 25% of patients undergoing THA to be anaemic before surgery [7]. Preoperative anaemia has been linked with higher rates of blood transfusion after surgery in multiple studies; however, there is  no Irish data to confirm this [8–11]. Groups have attempted to decrease transfusions after surgery such as THA by reversing preoperative anaemia through iron therapy before surgery [12], or by using measures in theatre to reduce blood loss during surgery [8, 9, 13, 14]. These have been largely met with positive results in terms of reduced patient morbidity, mortality, complications after surgery and therefore reduced hospital stay. Blood loss up to 1000ml may occur during THA surgery [15]. Internationally, blood transfusions due to THAs are common, occurring in approximately 16–25% of patients [16, 17].

Transfusions are independent risk factors for increased morbidity and mortality postoperatively [11, 14, 18, 19]. They are associated with increased length of stay in hospital [11, 14]. Blood transfusion risks include acute lung injury, graft versus host disease, multiorgan failure and death [20, 21], and make patients more vulnerable to infections such as pneumonia and sepsis [22]. Blood group mismatches are estimated to occur in approximately 5 patients per 100,000 transfusions [21]. Contaminated blood banks with HIV [23] and other viruses have occurred historically, infecting patients with negative consequences. Therefore, steps to reduce this risk to patients are not insignificant.

Blood is a valuable and scarce resource [17]. Blood is required daily in the Irish healthcare setting, with approximately 3000 blood donors needed weekly in Ireland [24]. Blood is also expensive, with a single unit costing hundreds of euro to transfuse [25]. Decreased transfusion rates would lead to significant financial savings.

## Study aims

The study is aimed at assessing preoperative factors associated with an increased incidence of postoperative blood transfusions, in patients who undergo total hip arthroplasty.

## Study objectives

The primary objective is determining the prevalence of preoperative anaemia in our sample population of patients undergoing total hip arthroplasty in South Infirmary-Victoria University Hospital, Cork, Ireland, and whether preoperative anaemia is associated with increased blood transfusion.

## Methods

### Study design and participants

This study was a retrospective cohort study, of all patients in the South Infirmary-Victoria University Hospital in Cork, Ireland, who underwent elective THA in 2019. The hospital database was accessed during November 2020 in the surgical ward of the hospital and used to identify patients who underwent THA from January 1, 2019, to December 31, 2019, for inclusion in the study. This time period was selected as it was a full calendar year of data, was the most recent data available for an entire year, and not impacted by the COVID-19 pandemic. In total, 350 patients were identified as fulfilling the criteria. The files were then obtained from SIVUH medical records. Of the 350 patients identified, 291 patient files have been included in the study. A total of 59 files were excluded due to logistical reasons.

### Study measures

Cases were defined as those who were documented in the chart as having received RCCs after THA before discharge from hospital. Variables collected were patient age, sex, haemoglobin levels before and after surgery, height, weight, BMI, blood transfusion records, anaesthesia administered, route of administering anaesthesia, tranexamic acid use, ASA grade, type of hip surgery, length of hospital stay, surgeon name, duration of surgery and drugs administered during surgery.

The W.H.O. definition of anaemia was used throughout this study. Preoperative anaemia was based on the results of the most recent haemoglobin score, which was recorded in a 6-week period prior to surgery during the preoperative assessment. Postoperative anaemia was based on the haemoglobin level in the most recent sample of blood analysed from the patient after surgery, usually collected the next day. The ASA score was documented on the anaesthetic sheet by the anaesthetist performing the case and collected from this document. The decision to transfuse a unit of blood was a medical decision made by the relevant doctor looking after the patient at that time.

### Ethical approval

Ethical approval to conduct this study was sought from the Clinical Research Ethics Committee of the Cork Teaching Hospitals. The application to seek approval to conduct the study was sent on February 4, 2020, and was granted on February 27th with an amendment made on August 14th which was approved on August 20, 2020. Following ethical approval, a further application was sent to the risk management department in SIVUH, requesting permission to conduct the study in the hospital. This application was sent on September 14, 2020, and approval was granted on October 5, 2020, by the Board of Directors in SIVUH.

### Data analysis

All of the data collected was entered in IBM SPSS version 22.0 for Windows (SPSS, Chicago, Illinois, USA) for analysis. Continuous variables were analysed using descriptive statistics to calculate the mean, median, range and 95% confidence intervals. Categorical variables were analysed using frequency tables. Pearson’s correlation coefficient was performed to analyse the relationships that exist between the variables where appropriate. A correlation coefficient 0.2 was used to define statistical significance.

## Results

### Study cohort

The total sample number was 291, with a male predominance (Table 1). The median age for patients was 65 years, mean age was 64.79 (standard deviation 11.79, range 29 to 90): 63.24 (SD = 11.73) for men and 66.45 (SD = 11.66) for women. Median blood loss was 250 mls. Postoperative blood transfusions occurred in 3.1% of patients (*n* = 9). Sixteen units of blood were transfused between the nine patients. Mean length of stay in hospital after surgery ranged from 2 to 12 days with a median of 4. In total, 99.3% (*n* = 289) patients in our study were recorded as being given tranexamic acid during surgery. Mean ASA score had a median of 2.00. BMI ranged from 17.40 to 50.50 with a median of 29.01.

**Table 1:** Patient demographic and variables

	**All**
Number of patients, *n* (%)	291 (100)
Age (years)	64.8 ± 11.7
Female, *n* (%)	141 (48.5)
ASA grade	2 ± 0.5
Length of stay	4.2 ± 1.6
Postoperative anaemia, *n* (%)	182 (62.5)
BMI	29.3 ± 5.1
Intraoperative blood loss (mls)	282.4 ± 153.3
Preoperative anaemia, *n* (%)	26 (8.9)
Postoperative blood transfusion, *n* (%)	9 (3.1)

Data shown as % of all patients or mean ± SD

*ASA* American Society of Anesthesiologists score, *BMI* body mass index

### Preoperative anaemia

In this study, 8.9% (*n* = 26) of patients were anaemic preoperatively. Of the 26 patients who were anaemic preoperatively, 19.2% (*n* = 5) received postoperative blood transfusions. In comparison, of the 91.5% of patients who were not anaemic preoperatively, 1.5% (*n* = 4) received postoperative blood transfusions (Table 2). The Pearson correlation for preoperative anaemia and postoperative blood transfusion was 0.3. The odds ratio of having a postoperative blood transfusion if anaemic before surgery was 15.5 compared to those who are not anaemic before surgery (95% CI 3.88–62.24). The relative risk for the outcome postoperative blood transfusion was 12.74 (95% CI 3.64–44.54).

**Table 2:** Preoperative anaemia vs postoperative anaemia; patient demographic and variables

	**Preoperative anaemia**	**No preoperative anaemia**	***p* value**
Number of patients, *n* (%)	26 (8.9)	265 (91.1)	N/A
Age (years)	72.5 ± 7.9	64 ± 11.8	0.000461
Female, *n* (%)	11 (42.3)	130 (49.1)	0.513
ASA grade	2.2 ± 0.5	2 ± 0.5	0.041
Length of stay	4.8 ± 1.7	4.2 ± 1.6	0.052
Postoperative anaemia, *n* (%)	25 (96.2)	157 (59.3)	0.000184
BMI	29.1 ± 5	29.3 ± 5.1	0.804
Postoperative blood transfusion, *n* (%)	5 (19.2)	4 (1.5)	0.00000039705

Data shown as % of all patients or mean ± SD

*ASA* American Society of Anesthesiologists score, *BMI* body mass index

### Postoperative blood transfusions

Of the 182 patients who had postoperative anaemia, 5% (*n* = 9) received postoperative blood transfusions whilst 95% (*n* = 173) did not. All 9 patients who received postoperative blood transfusions were anaemic postoperatively, whilst none of the 109 patients who were not anaemic postoperatively received any postoperative blood transfusions (Table 3). Preoperative haemoglobin ranged from 9 to 13.1 g/dl (mean = 11.37) in patients who received a postoperative blood transfusion compared to 10.2–17.6 g/dl (mean = 13.74) in those who did not. Postoperative haemoglobin ranged from 6.9 to 10.3 g/dl (mean = 8.46) in patients who later received postoperative blood transfusions compared to 8.4–15.7 g/dl (mean = 11.68) in the non-transfused group.

**Table 3:** Postoperative blood transfusion vs no postoperative blood transfusion; patient demographic and variables

	**Postoperative blood transfusion**	**No postoperative blood transfusion**	***p* value**
Number of patients, *n* (%)	9 (3.1)	282 (96.9)	N/A
Age (years)	77.3 ± 5.6	64.4 ± 11.7	0.001103
Female, *n* (%)	6 (66.7)	135 (47.9)	0.268
ASA grade	2.6 ± 0.5	2 ± 0.5	0.000612
Length of stay	6.4 ± 2	4.2 ± 1.5	0.000016
Postoperative anaemia, *n* (%)	9 (100)	173 (61.3)	0.018
BMI	27.3 ± 5	29.4 ± 5.1	0.242
Preoperative anaemia, *n* (%)	5 (55.6)	21 (7.4)	0.00000039705

Data shown as % of all patients or mean ± SD

*ASA* American Society of Anesthesiologists score, *BMI* body mass index

Patients who underwent postoperative blood transfusions had a greater mean length of stay than those who did not (*r* = 0.25). The mean age in the postoperative blood transfusion group was greater than that in the non-transfused (*r* = 0.19). The mean ASA score in those who received postoperative blood transfusions was higher than that in the non-transfused group (*r* = 0.2).

### ASA grade

There was a positive correlation between increasing ASA scores and increased length of stay (*r* = 0.3) that was statistically significant (*p* value < 0.001). A statistically significant relationship was also found between increasing ASA score and BMI value for patients included in this study (*p* value = 0.023, *r* = 0.13).

### Postoperative anaemia

The mean intraoperative blood loss in those who were anaemic postoperatively was 297.62 mls (95% CI 275.17–320.06). This was higher than those who were not anaemic postoperatively, with a mean blood loss of 256.98 mls (95% CI 228.42–285.54, *p* value = 0.028, *r* = 0.128).

## Discussion

Our study has found a statistically significant relationship between preoperative anaemia and postoperative blood transfusions. There was a weak, positive, statistically significant Pearson correlation associating increased rates of postoperative blood transfusions in patients who are anaemic preoperatively compared to those who are not. Our study also found an odds ratio of 15.5 for those who are anaemic preoperatively to have a postoperative blood transfusion, compared to those who are not. Our data is the first Irish surgical data to confirm other international findings.

Our sample cohort was predominantly male at 51.5% compared to females. Previous studies usually consisted of a female majority, ranging from 59 to 61.3% [18, 26]. The mean age of the patients in our study was 64.8 years, which is consistent with previous literature, with the mean age ranging from 60.8 to 64 years [18, 26]. Preoperative anaemia was prevalent in our cohort, with 8.9% of patients being defined by the W.H.O. as being anaemic before they underwent surgery. This 8.9% is in contrast to other studies performed on different sample populations, where levels of preoperative anaemia were higher, ranging from 12.9 to 24.6% [14, 18]. Postoperative blood transfusions occurred in 3.1% of the total sample population in our study. This figure is in contrast to previous studies which had much higher rates of transfusion, up to 25.1% of patients [26]. The much lower rate of postoperative blood transfusion is most likely due to the advancements in practice which have been made and implemented in recent years. Such practices include patient optimisation prior to surgery, carefully stopping anticoagulant medication in line with local protocols, advancement in surgical equipment and technique, tranexamic acid and differing Hb thresholds for transfusion (in our institution: < 7 g/dl or 8 g/dl if cardiac disease or symptomatic).

A statistically significant association was found between preoperative and postoperative anaemia (*p* value < 0.001). Patients who were anaemic preoperative had an odds ratio of 17.2 of being anaemic postoperatively compared to those who were not. Preoperative anaemia was found in this study to be a more prevalent condition as patients age with a mean age of 72.5 years versus 64 years. A statistically significant association was found between age and rates of preoperative anaemia from our research. This supports previous literature which suggested that the incidence of anaemia increases with ageing [27]. Preoperative anaemia was also found to be associated with increasing ASA scores in our patient cohort.

Our study found no statistically significant associations between preoperative anaemia and BMI status, blood loss intraoperatively or length of stay. Previous literature had found associations between preoperative anaemia and increased length of stay [28], but our study did not support such findings.

There was a strong association between postoperative anaemia and postoperative blood transfusions, which is expected. This finding has also been made in other studies [28]. Blood transfusions have been found previously to be associated with increased length of hospital stay [11, 18], likely due to the increased postoperative complications [14]. Transfusions have also been associated with increased patient costs [11]. In this study, patients who underwent postoperative blood transfusions had a longer mean length of stay of 6.44 days compared to those who did not have postoperative blood transfusions, with a mean length of stay of 4.16 days.

Patients who received postoperative blood transfusions were found in this study to have higher mean ASA grades compared to those who did not. Previous literature has associated higher ASA scores with increased transfusion rates both during and after surgery [10], but other papers found no such correlation [8]. This is useful as this is a scoring system implemented preoperatively that has been suggested by data analysis to be strongly associated statistically with postoperative blood transfusions. Therefore, this scoring system may be capable of guiding clinicians as to the increased likelihood of a patient requiring a blood transfusion postoperatively. No patient with an ASA score of 1 received a postoperative blood transfusion in this study. In contrast, 1.82% of patients with an ASA score of 2 received a postoperative blood transfusion, whilst 13.89% of patients with an ASA score of 3 received a postoperative blood transfusion, which is a significant difference. A positive correlation was found between increasing ASA scores and length of hospital stay. A moderate, positive correlation was also found between increasing age and ASA score, which is consistent with previous studies [29].

Blood conservation strategies have been researched extensively in the past [30]. As a result, tranexamic acid use during surgery has become a key part of our practice. In our study, 99.3% of patients received tranexamic acid intraoperatively. Previous literature has demonstrated that tranexamic acid use in patients with preoperative anaemia was associated with increased haemoglobin levels after surgery [8]. Patients who do not receive tranexamic acid have a greater incidence of  transfusion [13]. As our sample of patients who did not have tranexamic acid was so small, we were unable to test these previous findings.

## Conclusion

Preoperative anaemia is a common condition in an Irish patient cohort undergoing total hip arthroplasty. Preoperative anaemia was associated with a significantly increased risk of postoperative blood transfusions after total hip arthroplasty. Further research is needed to establish if management strategies to increase preoperative haemoglobin levels are effective at reducing postoperative blood transfusions. Postoperative blood transfusion was uncommon in our study, occurring in only 3.1% of patients who underwent total hip arthroplasty. Whether our institution’s near universal use of tranexamic acid is responsible could not be studied in our population. Patients who are at an increased risk of requiring postoperative blood transfusions are associated with having a higher ASA score.

## Limitations

This is a retrospective cohort study, and whilst every effort has been made to eliminate the effect of confounders, some may remain. This research was carried out in elective patients in an elective hospital in Ireland. This patient demographic and the findings related to this demographic may not be comparable to another setting. In total, 59 patient charts were unavailable to be included in this study, which may have introduced bias. The decision to transfuse a unit of blood was the independent medical decision of the doctors looking after the various patients. Individual practice may vary with regard to transfusion criteria.

## Data Availability

Raw data if desired can be requested by contacting the lead author directly by email.
